# Effects of a combination of amlodipine and imipenem on 42 clinical isolates of Acinetobacter baumannii obtained from a teaching hospital in Guangzhou, China

**DOI:** 10.1186/1471-2334-13-548

**Published:** 2013-11-16

**Authors:** Yu jun Li, Chu zhi Pan, Zi wen Zhao, Zhu xiang Zhao, Hui ling Chen, Wei bo Lu

**Affiliations:** 1 Department of Respiratory Medicine, Guangzhou First People’s Hospital, Guangzhou Medical University, Panfu Road, Guangzhou, China; 2 Department of Hepatobiliary Surgery, the Third Affiliated Hospital of Sun Yat-sen University, Tian He Road, Guangzhou, China; 3 Department of Clinic Laboratory, Guangzhou First People’s Hospital, Guangzhou Medical University, Panfu Road, Guangzhou, China

**Keywords:** Acinetobacter baumannii, MLST, Imipenem, Amlodipine

## Abstract

**Background:**

The clonal spread of *Acinetobacter baumannii* is a global problem, and carbapenems, such as imipenem, remain the first-choice agent against *A. baumannii*. Using synergy to enhance the antibiotic activity of carbapenems could be useful. Here, amlodipine (AML) was tested alone and with imipenem against *A. baumannii* isolates.

**Methods:**

Forty-two isolates of *A. baumannii* were collected. Multilocus sequence typing (MLST) assessed the genetic relationship of the isolates. The resistance phenotypes were determined using disc diffusion. The minimum inhibitory concentrations (MICs) of the drugs were determined by broth microdilution. The combined effects of the drugs were determined by a checkerboard procedure. Metallo-β-lactamase (MBL) was determined using the MBL Etest.

**Results:**

Forty-two *A. baumannii* isolates were collected from 42 patients who were mostly older than 65 years and had long inpatient stays (≥7 days). *A. baumannii* was mostly recovered from the respiratory system (N = 35, 83.3%). Most patients (N = 27, 64.3%) received care in intensive care units (ICUs). Disc diffusion testing demonstrated that *A. baumannii* susceptibility to polymyxin B was 100%, while susceptibility to other antimicrobial agents was less than 30%, classifying the isolates into 10 MDR and 32 XDR strains. MLST grouped the *A. baumannii* isolates into 4 existing STs and 6 new STs. STn4 carried allele G1, with a T → C mutation at nt3 on the gpi111 locus. STn5 carried allele A1, possessing A → C mutations at nt156 and nt159 on the gltA1 locus. ST195 and ST208 accounted for 68.05% (29/42) of the isolates. Clonal relation analysis showed that ST195 and ST208 belonged to clonal complex (CC) 92. The inhibitory concentration of imipenem ranged from 0.5 to 32 μg/ml, and that of AML ranged from 40 to 320 μg/ml. In combination, the susceptibility rate of *A. baumannii* isolates increased from 16.7% to 54.8% (P = 0.001). In the checkerboard procedure, half of the isolates (N = 21, 50.0%) demonstrated synergy or partial synergy with the drug combination. The MBL Etest revealed that 1 *A. baumannii* strain (N = 1, 2.4%) produced MBL.

**Conclusions:**

CC92 was the major clone spreading in our hospital. AML improved the activity of imipenem against *A. baumannii* isolates in vitro but did not inhibit MBL.

## Background

*Acinetobacter baumannii* (AB) has emerged as one of the most troublesome pathogens in health care institutions globally. It is characterised by frequent episodes of multi-drug resistance due to multiple mechanisms, and therapeutic options have become increasingly restricted [1-3]. Unfortunately, the present rate of discovery of antibiotics is much slower than that in the past [4]. This problem is exemplified by the fact that it is necessary to identify potential reservoirs of the organism and modes of transmission to control the spread of *A. baumannii* in hospitals. To distinguish the outbreak strain from epidemiologically unrelated acinetobacters, a comparison of isolates at the subspecies level is required. Multilocus sequence typing (MLST) is a highly discriminative typing system in which typing data are translated into a numerical code that can be obtained in an identical manner at different laboratories using the same protocol. It provides a portable method that may be suitable for global epidemiologic studies and that may allow for the recognition of epidemic, multiresistant, and virulent *A. baumannii* clones and the monitoring of their national and international spread [1,5,6]. However, data have been limited in Guangzhou.

Recently, an effective approach has been to explore non-antibiotic compounds (helper compounds) that express antibacterial properties, possibly by acting through different mechanisms from those of existing drugs, either by the enhancement of antibiotic activity (synergism) or the reversal of antibiotic resistance, yielding sensitivity comparable to that derived from classical antibiotics in previously drug-resistant microorganisms [7]. Among these non-antibiotics, amlodipine (AML) is the most promising helper compound. It has been shown to exhibit in vitro activity against a wide range of bacteria [8-11], but few studies have reported its potentiating effects on clinical *A. baumannii* isolates. In this study, AML was tested, alone and in combination with imipenem, against *A. baumannii* isolates.

## Methods

### Bacterial isolates and drugs

A total of 42 isolates of *A. baumannii* were collected from patients treated at our hospitals. The Guangzhou First People’s Hospital Ethics Committee approved the protocol, and all of the patients provided informed consent for inclusion in the study. The total number of beds in the hospitals was 1571. All of the *A. baumannii* isolates were consecutively collected from April 2011 to February 2012 from clinical specimens (respiratory secretion, blood, cerebrospinal fluid, and urine) from patients who were hospitalised in the general wards and intensive care units. Duplicate isolates from the same patients were excluded. The isolates were identified using the Vitek 2 (bioMerieux, Inc. Durham, North Carolina, USA) automated microbiology system.

Antibiotic discs (OXOID) were obtained from Melone Pharmaceutical Co. Ltd. (China). AML was obtained from Melone Pharmaceutical Co. Ltd. (China), and imipenem was obtained from Merck & Co. Inc. Imipenem was dissolved in phosphate-buffered saline (PBS) according to CLSI (M100-S22, 2012) [12]. AML was dissolved in dimethyl sulfoxide (DMSO) [9].

### Multilocus sequence typing (MLST)

MLST was performed on *A. baumannii,* as described by Bartual et al. [5]. Briefly, seven housekeeping genes, i.e., gltA, gyrB, gdhB, recA, cpn60, gpi, and rpoD, were amplified, followed by sequencing on the ABI Prism Sequencer 3730 (Applied Biosystems). The sequences of these seven housekeeping genes were analysed using the Pubmlst database [13]. The sequence type (ST) was designated according to the allelic profiles in the database. The eBURST algorithm (version 3) [14] was used to assign STs to CCs and to assess the genetic relationship with the definition of the groups sharing alleles at ≥6 of 7 loci. The CC comprises a founding ST as a common ancestor, as well as several other closely related STs descending from the predicted founding genotype.

### Antimicrobial susceptibility testing and metallo-β-lactamase detection

The strains were stored at −80°C in trypticase soy broth supplemented with 15% glycerol and were passaged twice on a blood agar plate before testing. *Escherichia coli* ATCC 25922 and *Pseudomonas aeruginosa* ATCC 27853 were used as the control organisms.

All 42 of the isolates were tested against a panel of antibiotics with the disc diffusion method, as recommended by the Clinical and Laboratory Standards Institute (CLSI; M100-S22, 2012) [12], to determine the resistance phenotype. Multidrug resistance (MDR) was defined as acquired non-susceptibility to at least one agent in three or more antimicrobial categories, and extensive drug resistance (XDR) was defined as non-susceptibility to at least one agent in all but two or fewer antimicrobial categories (i.e., bacterial isolates remaining susceptible to only one or two categories) [3]. The MBL Etest (bioMerieux, Inc. Solna, Sweden) was applied to detect metallo-β-lactamase (MBL) [15].

The MIC of AML was defined as the lowest concentration at which no growth was observed. All of the broth preparations used in the microdilution testing and the Mueller Hinton broth were freshly prepared and used within 24 hours. The checkerboard procedure [10] was used to determine the combined effects of AML and imipenem. Synergy was determined by calculating the fractional inhibitory concentration (FIC) index as follows: FIC index = FICA + FICB = [A]/MICA + [B]/MICB, where [A] is the concentration of drug A; FICA is the FIC of drug A; and [B], MICB, and FICB are defined in the same manner for drug B. The FIC index was interpreted as follows: <0.5, synergy; 0.5 to 0.75, partial synergy; 0.76 to 1.0, additive effect; >1.0 to 4.0, indifference; and >4.0, antagonism.

### Statistical analysis

SPSS (version 19.0) was used for all of the calculations. Percentages were compared using the chi-square test. A P value ≤0.05 in a two-tailed test was considered to indicate statistical significance.

## Results

### Summary of 42 A. baumannii isolates and MLST

A total of 42 *A.* baumannii isolates were collected from 42 patients; 30 patients were male, and 12 were female. Most of the patients were older than 65 years and had long inpatient stays (≥7 days). The most common site from which *A. baumannii* was recovered was the respiratory system (N = 35, 83.3%), followed by the blood (N = 3, 7.1%). Most of the patients (N = 27, 64.3%) were receiving care in intensive care units (ICUs), while 15 were in general wards (see Table 1).

**Table 1:** **Summary of 42 *A. baumannii *isolates**

**Gender (%)**	**Age (%)**	**Hospital ward (%)**	**Type of specimen (%)**	**Inpatient status (%)**
Male 30 (71.4%)	≤17 0 (0%)	ICU 27 (64.3%)	Respiratory 35 (83.3%)	≤2 days 4 (9.5%)
Female 12 (28.6%)	18-65 9 (21.4%)	Medical 10 (23.8%)	Blood 3 (7.1%)	3-6 days 9 (21.4%)
	≥66 33 (78.6%)	Surgical 4 (9.5%)	Wound 2 (4.8%)	≥7 days 29 (69.1%)
		Burn 1 (2.4%)	Urine 1 (2.4%)	
			CSF 1 (2.4%)	

ICU, intensive care unit; CSF, cerebrospinal fluid.

**Figure 1:**
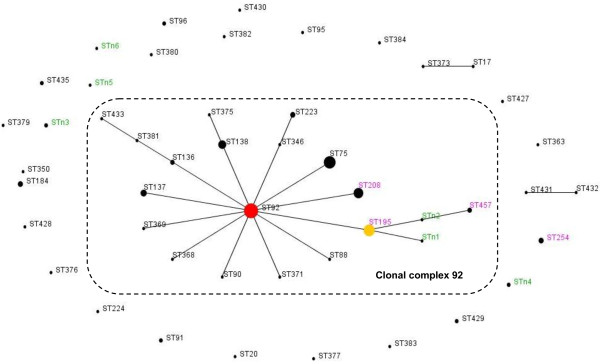
**Population snapshot of *A. baumannii *in this study and existing isolates in China.** Population snapshot of *A. baumannii* in this study and existing isolates in China, based on the data contained in the Pubmlst database [13] as of 27 April 2013, represented by an eBURST algorithm. Circles represent STs, and their sizes correspond to the number of isolates. The red circle represents the founder ST (ST92). The broken line indicates clonal complex (CC) 92. The ST labels are coloured as follows: black, STs found only in the Pubmlst database; green, STs found only in this study; and purple, STs found in both the Pubmlst database and this study. STn3, STn4, STn5, STn6, and ST254 were the singletons in this study.

According to the MLST, the 42 *A. baumannii* isolates could be grouped into four existing STs [Additional file 1] (ST195, 1-3-3-2-2-96-3; ST208, 1-3-3-2-2-97-3; ST254, 21-15-3-2-35-111-4; and ST457, 1-15-3-2-2-153-3) and six new STs (STn1, 1-3-3-2-2-96-5; STn2, 1-15-3-2-2-96-3; STn3, 1-81-3-2-2-96-4; STn4, 21-15-3-2-35-G1-3; STn5, A1-15-3-2-2-153-4; and STn6, 11-65-3-20-37-96-15). STn4 carried a new allele, G1, which had a T → C mutation at nt3 of the gpi111 locus. STn5 had a new allele, A1, that had two mutations at the gltA1 locus, which were A → C mutations at nt156 and nt159. All of the isolates belonging to ST195 and ST208 accounted for 68.05% (29/42) of the isolates. Clonal relation analysis showed that both ST195 and ST208 belonged to the globally disseminated CC92 for *A. baumannii* (see Table 2 and Figure 1).

**Table 2:** Multilocus sequence typing of 42 isolates

**Number of isolates**	**Specimens**	**Wards**	**gltA**	**gyrB**	**gdhB**	**recA**	**cpn60**	**gpi**	**rpoD**	**ST**
1	Sputum	ICU	1	3	3	2	2	97	3	ST208
2	Blood	ICU	1	3	3	2	2	96	5	STn1
3	Sputum	ICU	1	3	3	2	2	97	3	ST208
4	Blood	ICU	1	3	3	2	2	96	3	ST195
5	BALF	RICU	1	3	3	2	2	96	3	ST195
6	Sputum	RICU	1	3	3	2	2	96	3	ST195
7	Sputum	Respiratory	1	15	3	2	2	153	3	ST457
8	Sputum	RICU	1	3	3	2	2	96	3	ST195
9	Sputum	ICU	1	3	3	2	2	96	3	ST195
10	Sputum	Respiratory	1	3	3	2	2	97	3	ST208
11	Sputum	Neurosurgery	1	15	3	2	2	96	3	STn2
12	Sputum	RICU	1	3	3	2	2	96	3	ST195
13	Sputum	Respiratory	1	3	3	2	2	97	3	ST208
14	Sputum	ICU	1	3	3	2	2	96	3	ST195
15	BALF	RICU	1	3	3	2	2	96	3	ST195
16	Sputum	ICU	1	3	3	2	2	96	3	ST195
17	Wound	Gastroenterology	1	3	3	2	2	96	3	ST195
18	Sputum	Respiratory	1	3	3	2	2	97	3	ST208
19	Sputum	ICU	1	3	3	2	2	97	3	ST208
20	Urine	Geriatrics ICU	21	15	3	2	35	111	4	ST254
21	BALF	RICU	1	3	3	2	2	96	3	ST195
22	BALF	RICU	1	3	3	2	2	96	3	ST195
23	Sputum	Respiratory	1	3	3	2	2	97	3	ST208
24	Sputum	ICU	1	3	3	2	2	97	3	ST208
25	Sputum	RICU	1	3	3	2	2	96	3	ST195
26	Sputum	RICU	1	3	3	2	2	96	3	ST195
27	Sputum	Neurosurgery	1	81	3	2	2	96	4	STn3
28	Sputum	Respiratory	21	15	3	2	35	111	4	ST254
29	Sputum	RICU	21	15	3	2	35	111	4	ST254
30	Sputum	RICU	1	3	3	2	2	96	3	ST195
31	Sputum	Respiratory	1	3	3	2	2	97	3	ST208
32	Sputum	Neurosurgery	1	81	3	2	2	96	4	STn3
33	Sputum	ICU	1	3	3	2	2	96	3	ST195
34	BALF	RICU	1	15	3	2	2	153	3	ST457
35	CSF	ICU	1	3	3	2	2	96	3	ST195
36	Sputum	Respiratory	21	15	3	2	35	G1	3	STn4
37	Sputum	ICU	1	3	3	2	2	96	3	ST195
38	Wound	Burn	A1	15	3	2	2	153	4	STn5
39	Sputum	Geriatrics ICU	21	15	3	2	35	G1	3	STn4
40	Blood	Urinary surgery	1	3	3	2	2	96	3	ST195
41	Sputum	Nephrology	1	3	3	2	2	97	3	ST208
42	BALF	RICU	11	65	3	20	37	96	15	STn6

BALF, bronchoalveolar lavage fluid; CSF, cerebrospinal fluid; ICU, intensive care unit; RICU, respiratory intensive care unit; G1, a new allele that has a T → C mutation at nt3 in the gpi111 locus; A1, a new allele possessing two mutations at the gltA1 locus (A → C mutations at nt156 and nt159).

### Antimicrobial susceptibility testing and metallo-β-lactamase detection

The data from the disc diffusion testing (see Table 3) demonstrated that the susceptibility of *A. baumannii* to polymyxin B was 100%, while its susceptibility to other antimicrobial agents was lower than 30%, thus classifying the 42 *A. baumannii* isolates into 10 MDR and 32 XDR strains. In particular, no *A. baumannii* isolates were susceptible to ceftazidime, ceftriaxone, cefepime, aztreonam, levofloxacin, ciprofloxacin, or doxycycline, which have been routinely used in clinical practice in China. Only one *A. baumannii* strain (N = 1, 2.4%) demonstrated MBL production, according to the MBL Etest.

**Table 3:** Isolates were tested against antibiotics by disc diffusion

**Isolate no.**	**SCF**	**TZP**	**CAZ**	**CRO**	**FEP**	**ATM**	**IPM**	**MEM**	**LEV**	**CIP**	**AK**	**DO**	**TGC**	**PB**	**TOB**	**Phenotype/MBL**
1	R	R	R	R	R	R	R	R	R	R	R	R	S	S	R	XDR/Neg
2	R	R	R	R	R	R	R	R	R	R	R	R	S	S	R	XDR/Neg
3	R	R	R	R	R	R	R	R	R	R	R	R	S	S	R	XDR/Neg
4	R	R	R	R	R	R	R	R	R	R	R	R	I	S	R	XDR/Neg
5	R	R	R	R	R	R	R	R	R	R	R	R	I	S	R	XDR/Neg
6	R	R	R	R	R	R	R	R	R	R	S	R	I	S	S	MDR/Neg
7	R	R	R	R	R	R	R	R	R	R	R	R	I	S	R	XDR/Neg
8	R	R	R	R	R	R	R	R	R	R	R	R	I	S	R	XDR/Neg
9	R	R	R	R	R	R	R	R	R	R	R	R	I	S	R	XDR/Neg
10	R	R	R	R	R	R	R	R	R	R	R	R	I	S	R	XDR/Neg
11	R	R	R	R	R	R	R	R	R	R	R	R	I	S	R	XDR/Neg
12	R	R	R	R	R	R	R	R	R	R	S	R	I	S	S	MDR/Neg
13	R	R	R	R	R	R	R	R	R	R	R	R	I	S	R	XDR/Neg
14	S	R	R	R	R	R	I	I	R	R	R	R	S	S	R	MDR/Neg
15	R	R	R	R	R	R	R	R	R	R	R	R	I	S	R	XDR/Neg
16	R	R	R	R	R	R	R	R	R	R	R	R	R	S	R	XDR/Neg
17	R	R	R	R	R	R	R	R	R	R	R	R	I	S	R	XDR/Neg
18	R	R	R	R	R	R	R	R	R	R	R	R	I	S	R	XDR/Neg
19	R	R	R	R	R	R	R	R	R	R	R	R	I	S	R	XDR/Neg
20	I	R	R	R	R	R	R	R	R	R	R	I	S	S	R	XDR/Neg
21	R	R	R	R	R	R	R	R	R	R	R	R	R	S	R	XDR/Neg
22	R	R	R	R	R	R	R	R	R	R	S	R	R	S	S	MDR/Neg
23	I	R	R	R	R	R	R	R	R	R	R	R	S	S	R	XDR/Neg
24	S	R	R	R	R	R	S	S	R	R	S	R	I	S	S	MDR/Neg
25	R	R	R	R	R	R	R	R	R	R	R	R	S	S	R	XDR/Neg
26	R	R	R	R	R	R	R	R	R	R	R	R	S	S	R	XDR/Neg
27	R	R	R	R	R	R	S	I	R	R	R	R	I	S	R	MDR/Neg
28	I	R	R	R	R	R	R	R	R	R	R	R	S	S	R	XDR/Neg
29	I	R	R	R	R	R	R	R	R	R	R	I	S	S	R	XDR/Neg
30	R	R	R	R	R	R	R	R	R	R	R	R	I	S	R	XDR/Neg
31	R	R	R	R	R	R	R	R	R	R	R	R	I	S	R	XDR/Neg
32	I	R	R	R	I	R	S	S	R	R	R	I	I	S	R	MDR/Neg
33	R	R	R	R	R	R	R	R	R	R	R	R	I	S	R	XDR/Neg
34	R	R	R	R	R	R	R	R	R	R	R	R	I	S	R	XDR/Neg
35	R	R	R	R	R	R	R	R	R	R	R	R	I	S	R	XDR/Neg
36	I	R	R	R	R	I	R	R	R	R	R	S	S	S	R	MDR/Neg
37	R	R	R	R	R	R	R	R	R	R	R	R	I	S	R	XDR/Neg
38	R	R	R	R	R	R	R	R	R	R	R	R	I	S	R	XDR/Neg
39	R	R	R	R	R	R	R	R	R	R	R	I	S	S	R	XDR/Pos
40	R	R	R	R	R	R	R	R	R	R	R	R	I	S	R	XDR/Neg
41	I	R	R	R	R	R	S	I	R	R	R	R	R	S	R	MDR/Neg
42	I	S	R	R	R	R	S	S	I	R	R	I	R	S	R	MDR/Neg
Susceptible (%)	2.4	2.4	0.0	0.0	0.0	0.0	11.9	7.1	0.0	0.0	9.5	0.0	28.6	100.0	9.5	-

Metallo-β-lactamase was detected by MBL Etest.

R, resistance; S, sensitivity; I, intermediate; XDR, extensively drug-resistant; MDR, multidrug-resistant; MBL, metallo-β-lactamase; Pos, positive; Neg, negative; PB, polymyxin B (300 units); TGC, tigecycline (15 μg); IPM, imipenem (10 μg); AK, amikacin (30 μg); TOB, tobramycin (10 μg); MEM, meropenem (10 μg); TZP, piperacillin/tazobactam (100/10 μg); SCF, cefoperazone/sulbactam (70/35 μg); CAZ, ceftazidime (30 μg); CRO, ceftriaxone (30 μg); FEP, cefepime (30 μg); ATM, aztreonam (30 μg); LEV, levofloxacin (5 μg); CIP, ciprofloxacin (5 μg); DO, doxycycline (30 μg).

**Figure 2:**
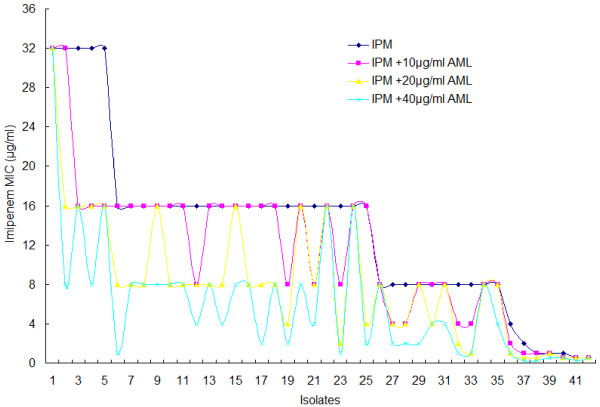
**The change in the imipenem MIC by adding AML.** Figure 2 shows the distribution of imipenem MICs. The change in the imipenem MIC with the addition of 10, 20, and 40 μg/ml of AML compared with the MIC resulting from no AML addition. The imipenem MICs decreased significantly with the addition of 40 μg/ml of AML.

Table 4 shows the resistance reversal effects in *A. baumannii* isolates tested with imipenem. The inhibitory concentrations of imipenem alone against all of the strains tested ranged from 0.5 to 32 μg/ml, and the AML inhibitory concentrations ranged from 40 to 320 μg/ml. In combination with AML, the susceptibility rate of *A. baumannii* isolates to imipenem increased from 16.7% to 54.8% (P = 0.001). Figure 2 clearly shows the effects on imipenem MIC changes when different concentrations of AML were added. The imipenem MICs decreased significantly with the addition of 40 μg/ml of AML. Note that the resistance reversal by AML in combination with imipenem occurred at concentrations that were much lower than the MIC_90_ required for the growth inhibition of 90% of *A. baumannii* isolates by the compound.

**Table 4:** **Imipenem potency in the absence and presence of AML in 42 *A. baumannii *isolates**

**Compound/combination of compounds**	**MIC (μg/ml)**			**No. susceptible (%)**	**No. nonsusceptible (%)**^**a**^	**Statistical analysis**^**b**^	
	**MIC**_**Range**_	**MIC**_**50**_	**MIC**_**90**_			**χ**^**2 **^**-values**	**P values**
AML	40-320	80	160	-	-	-	-
IPM	0.5-32	16	16	7 (16.7%)	35 (83.3%)	-	-
IPM +10 μg/ml AML	0.5-32	8	16	13 (31.0%)	29 (69.0%)	2.363	0.200
IPM +20 μg/ml AML	0.5-32	8	16	15 (35.7%)	27 (64.3%)	3.941	0.081
IPM +40 μg/ml AML	<0.5-32	4	16	23 (54.8%)	19 (45.2%)	13.274	0.001

MIC, minimum inhibitory concentration; MIC_50_ and MIC_90_, MICs for 50% and 90% of strains, respectively; ^a^nonsusceptible, including intermediate resistance; ^b^statistical analysis compared with the IPM group.

In synergy testing with the checkerboard procedure (see Table 5), half of the isolates (N = 21, 50.0%) demonstrated synergy or partial synergy with the imipenem and AML combination. The FIC index values ranged from 0.375 to 0.75. Ten isolates (N = 10, 23.8%) demonstrated additive effects; 11 (26.2%) isolates demonstrated indifference; and no cases of antagonism were observed.

**Table 5:** **Fractional inhibitory concentration (FIC) index of the checkerboard procedure in 42 *A. baumannii *isolates**

**Combination of compounds**	**FIC index (N,%)**				
	**<0.5 Synergy**	**0.5 to 0.75 partial synergy**	**0.76 to 1.0 additive effect**	**>1.0 to 4.0 indifference**	**>4.0 antagonism**
Imipenem + AML	6 (14.3%)	15 (35.7%)	10 (23.8%)	11 (26.2%)	0 (0%)

The concentrations of imipenem and AML used in the checkerboard procedure depended on the MICs of each *A. baumannii* isolate tested.

## Discussion

*A. baumannii* infections have become more difficult to treat due to the emergence of isolates that are resistant to multiple antimicrobial drugs. Data from the Chinese Network for Bacterial Resistance Surveillance demonstrated that almost 17% of these isolates were resistant to all of the antimicrobial agents that are routinely used in clinical practice and that the susceptibility of *A. baumannii* to carbapenems has decreased to less than 50% [16]. As a consequence, infections caused by *A. baumannii* strains have been associated with high mortality and treatment failure [17]. According to our data, *A. baumannii* has been widely distributed in our hospital and has been isolated from the respiratory system, blood, wounds, urine, and cerebrospinal fluid, especially from ICU patients who are older and have longer inpatient stays, similar to the results reported in many other studies [1,18-20]. The susceptibility of these isolates to imipenem was 16.1% (according to the MIC method). Only 1 (2.4%) MBL-producing *A. baumannii* strain was found in our study, which was similar to the findings of Hui Wang [21], in which only 1 isolate among 221 nonrepetitive imipenem-resistant clinical isolates of *Acinetobacter* spp*.* carried the bla(IMP-8) MBL gene. The present study demonstrated that MBL was not the primary cause of *A. baumannii* resistance to imipenem in our hospital. Class D β-lactamases, the modification of penicillin-binding proteins and porins, or the upregulation of the AdeABC efflux system may have played more important roles in their potential resistance mechanisms [22]; thus, further investigation is needed.

MLST is an unambiguous typing method that has achieved notable success in global epidemiological investigations of *A. baumannii*[5,6]. Recently, some studies [6,23-26] have revealed that CC92 (previously known as CC22) has worldwide dissemination. The ST92 *A. baumannii* isolate is the most common clone, and it has been predicted to be the founder of this clonal complex. Yiqi Fu et al. [26] confirmed that CC92 represented the most epidemic STs in China. In addition to ST92, the other STs belong to CC92 and vary by area. A study by He et al. [25] showed that more ST138 isolates are associated with ST92 in western China. However, results from Qiao Zhong et al. [23] indicated that ST75, accompanied by ST92, may be the most common epidemical sequence types in eastern China. In our study, we assumed that ST195 and ST208 may be more common in southern China. These differences may be caused by different antibiotic usage habits, which could have influenced the evolutionary direction of ST92. Why has this clonal complex been so successful? Studies have shown that antibiotic susceptibility of CC92 is variable [27]. These findings may suggest that adaptation to the hospital environment, as well as antibiotic resistance, has been more important for the success of *A. baumannii* as a nosocomial pathogen.

Because imipenem remains the first-choice agent in China against *A. baumannii* strains [28], finding a way to enhance the antibiotic activity (synergism) of carbapenems against *A. baumannii* strains could be encouraging. Calcium channel blockers (CCBs) are a heterogeneous class of drugs used for the control of hypertension, angina, and cardiac arrhythmias [29]. However, many studies have reported a wide variety of broad-spectrum antibacterial activities of CCBs [30], the most powerful of which was AML. Kumar [9] reported in vitro antimicrobial activity of AML against several Gram-positive and Gram-negative standard and clinical strains, including *Bacillus pumilus* NCTC 8241, *Staphylococcus aureus* NCTC 6571, *Escherichia coli* K12Row, *Salmonella typhimurium* NCTC74, *Shigella dysenteriae* 7 NCTC 519/66, *Shigella sonnei* 1NCTC 5/59, *Shigella flexneri* 4a24, *Shigella boydii* 8 NCTC 254/66, *Klebsiella pneumoniae* 14, *Vibrio cholerae* ATCC 14033, and *P. aeruginosa* APC. The inhibitory concentrations of AML against most of the strains tested ranged from 25 to 200 μg/ml, but its effects on *P. aeruginosa*, *E. coli*, and *K. pneumoniae* were limited, and the inhibitory concentrations were >800, >800, and 400 μg/ml, respectively. A further study demonstrated that AML in combination with streptomycin had a synergistic effect [10]. In addition to its in vitro and in vivo activity against Gram-positive and Gram-negative bacteria, AML showed a two- to eightfold reduction in its MIC when combined with streptomycin, and vice versa [10]. Similarly, when paired with levofloxacin, AML showed potential synergistic effects in the eradication of *P. aeruginosa* biofilms [11]. Our research demonstrated the antimicrobial activities of AML against clinical *A. baumannii* strains, and the MICs ranged from 40 to 320 μg/ml. In combination with imipenem, half of the isolates (N = 21, 50.0%) demonstrated synergy or partial synergy. Further investigations, such as the development of a time-killing curve, which may demonstrate killing synergistic effects against *A. baumannii* strains, are needed.

To date, research on the antimicrobial efficacy of AML has been conducted only in vitro and in animal models. Chronic toxicity tests with healthy mice showed that the therapeutic dose of AML (10 mg/kg) was within a safe and acceptable margin. Low doses of AML at 1.5 mg/kg/day continue to show good efficacy in mice [7]. According to our study results, AML must be used at doses of up to 40 μg/ml to improve the activity of imipenem against *A. baumannii* isolates. However, in humans, the recommended dose of AML is much lower, at only 10 mg/day (Cmax 6.14 ng/ml) as an antihypertensive agent, thus currently limiting the clinical application of AML [31]. However, AML has been able to cure highly virulent bacterial infections in mice. This ability suggests that AML could be employed as a ‘lead compound’ to synthesise more active novel agents that may be free of side effects, such as hypotension. Because both drugs have been satisfactorily used for a long time in clinical medicine for different purposes, their combination in humans is feasible.

## Conclusions

CC92 was the major clone that spread in our hospital. AML improved the activity of imipenem against *A. baumannii* isolates in vitro, but it did not work by inhibiting MBL.

## Competing interests

The authors declare that they have no competing interests.

## Authors’ contributions

YjL supervised the study, performed the susceptibility testing and metallo-β-lactamase detecting, and wrote the manuscript. ZxZ and WbL contributed to the susceptibility testing and metallo-β-lactamase detection. HlC provided advice regarding the susceptibility testing technology. CzP discussed the data and helped to finalise the manuscript. ZwZ planned and supervised the experiments. All authors read and approved the final manuscript.

## Pre-publication history

The pre-publication history for this paper can be accessed here:

http://www.biomedcentral.com/1471-2334/13/548/prepub

## Supplementary Material

Additional file 1 **Summary of 125 existing *A. baumannii *isolates in China.** The file’s format is excel spreadsheet, the file contains data of 125 existing *A. baumannii* isolates in China, which were downloaded from the Pubmlst database (http://pubmlst.org/abaumannii/), up to 27 April 2013. Click here for file
